# Dimensional Changes of Lumbar Intervertebral Foramen in Direct Anterior Approach‐Specific Hyperextension Supine Position

**DOI:** 10.1111/os.12728

**Published:** 2020-06-28

**Authors:** Ming‐yang Liu, Hai‐bo Wang, Shi‐wei Liu, Guan‐peng Zhang, Jian‐guo Liu, Chen Yang

**Affiliations:** ^1^ Department of Orthopaedic Surgery The First Hospital of Jilin University Changchun China

**Keywords:** Direct anterior approach, Hyperextension, Lumbar intervertebral foramen, Total hip arthroplasty

## Abstract

**Objective:**

To investigate the changes in the lumbar intervertebral foramen (LIVF) dimensions from neutral supine to direct anterior approach (DAA)‐specific hyperextension supine position through a standardized three‐dimensional (3D) reconstruction computerized tomography (CT) method.

**Methods:**

A total of 35 healthy volunteers (18 men and 17 women) were included in this retrospective study. The mean age of enrolled subjects was 28.9 ± 5.0 years. In September 2018, all the individuals underwent a 3D CT scan of the lumbar spine in neutral and 30° hyperextension supine positions, which mimicked the patient’s position in DAA total hip arthroplasty (THA). The dimensions of the LIVF, including foraminal area, height, and width, were measured on 3D reconstructed CT models at all lumbar foraminal levels. Foraminal area was defined as the area bounded by the adjacent superior and inferior vertebral pedicles, the posterosuperior boundary of the inferior vertebral body, the surface of the intervertebral disc posteriorly, the posteroinferior boundary of the superior vertebral body, and the surface of the ligamentum flavum anteriorly. Foraminal height was defined as the longest distance between the border of the superior and the inferior pedicle. Foraminal width was defined as the shortest distance between the posteroinferior edge of the superior vertebrae and the opposing boundary. Subgroup analysis and multiple linear regression were used to evaluate the relationship between percentage changes of the LIVF dimensions and side, sex, and age.

**Results:**

The LIVF dimensions varied significantly between the two positions at all levels (*P* < 0.05). From neutral to hyperextension supine position, the foraminal area reduced by 20.1% at lumbar 1–2 (L_1–2_), 22.6% at L_2–3_, 19.9% at L_3–4_, 18.1% at L_4–5_, and 12.0% at lumbar 5–sacral 1 (L_5_–S_1_) level, respectively; the foraminal height reduced by 9.5% at L_1–2_, 10.5% at L_2–3_, 9.5% at L_3–4_, 9.6% at L_4–5_, and 6.1% at L_5_–S_1_ level, respectively; the foraminal width reduced by 12.8% at L_1–2_, 14.5% at L_2–3_, 13.0% at L_3–4_, 10.4% at L_4–5_, and 8.4% at L_5_–S_1_ level, respectively. The changes in LIVF dimensions were biggest at L_2–3_ level and smallest at L_5_–S_1_ level. Subgroup analysis showed that there were no significant differences in the percentage changes of LIVF dimensions between the sexes and sides (*P* > 0.05). Multiple linear analysis showed that the percentage changes of LIVF dimensions were not related to side, sex, and age (*P* > 0.05).

**Conclusion:**

The dimensions of the LIVF showed significant decrease at all levels in the DAA‐specific hyperextension supine position compared with the neutral supine position, and the percentage changes of LIVF dimensions were not influenced by side, sex, and age.

## Introduction

Total hip arthroplasty (THA) is a reliable and effective surgical intervention for patients with end‐stage hip joint disease^1^, ^2^. The posterolateral approach, the lateral approach, and the direct anterior approach (DAA) are the most commonly used approaches during the operation^3^, ^4^. DAA is a muscle‐sparing method that provides a truly intranervous and intermuscular approach^5^, ^6^. It has gained increasing popularity due to less soft tissue damage and shorter recovery time ^3^, ^4^, ^7^. The exposure of the proximal femur is the key step in the DAA operation. Typically, the patient is placed in supine position and the hip is centered over the hinge of the operating table at the level of the anterior superior iliac spine (ASIS)^3^. The table can be extended at 30° during the operation to simplify the exposure of the femur^3^, ^7^.

However, we questioned whether this procedure is harmless to the patients. In our experience, some patients have complained about lumbar pain after the DAA THA, although it was relieved later. A recent publication reported that the hyperextension impact could lead to a chance fracture. This indicates that position‐related complications do exist in DAA THA^8^. We also wanted to find out whether this hyperextension position impacts the lumbar intervertebral foramen (LIVF), the nerve root outlet area. The boundary of the LIVF consists of adjacent superior and inferior vertebral pedicles, the posterosuperior margin of the inferior vertebral body, the intervertebral disc, the posteroinferior margin of the superior vertebral body, the superior and inferior articular facets and the ligamentum flavum^9^, ^10^, ^11^. Theoretically, the dimensions of the LIVF decrease from flexion to extension positions^11^, ^12^, ^13^, ^14^, ^15^. Extension can increase the facet joint movement and the bulging of the intervertebral disc and ligamentum flavum, resulting in more contact with the nerve root and a higher chance of compression or irritation of the nerve root^16^. Therefore, the specific position in the DAA THA might be a possible risk factor for nerve root injury, especially with prolonged operation time in a continuous hyperextension position.

Previous studies have elucidated the dimensional changes of LIVF from flexion to extension in standing or sitting position. Ren *et al*.^12^ discovered that the foraminal area, height, and width decreased significantly at lumbar 1–5 (L_1–5_) levels from neutral to extension standing position in patients with low back pain, while they did not find changes in LIVF dimensions at the lumbar 5–sacral 1 (L_5_–S_1_) level. In another *in vivo* dynamic study, Zhong *et al*.^13^ measured the LIVF dimensions from a flexion position of 45° to a maximal extension standing position with weight‐lifting activity in asymptomatic volunteers. They found that the foraminal area and width decreased significantly at all lumbar foraminal levels except L_5_–S_1_, and that the foraminal height remained constant throughout the activity. However, another study by Singh *et al*.^11^ compared the foraminal area under flexion standing, upright sitting, and extension standing positions in patients with low back pain. They found that the foraminal area increased significantly from flexion standing to upright sitting position at all lumbar foraminal levels except L_5_–S_1_ but decreased significantly from upright sitting to extension standing position at all levels, including L_5_–S_1_.

In addition, they found that the decrease in the foraminal area was biggest at the L_2–3_ level and smallest at the L_5_–S_1_ level. Similarly, Schmid *et al*.^14^ reported that the foraminal area decreased significantly from upright sitting to extension supine position in asymptomatic volunteers at all lumbar foraminal levels, including L_5_–S_1_. Furthermore, Schmid *et al*.^14^ and Zamani *et al*.^15^ both found a decrease in the foraminal area in extension sitting position and an increase in flexion sitting position at all levels. However, detailed information regarding the morphological changes of the LIVF from conventional neutral supine position to DAA‐specific hyperextension supine position has not been reported.

Knowing the dimensional changes of LIVF from neutral to hyperextension supine position may provide valuable information to understand the potential nerve root complications after DAA THA. In this study, the LIVF dimensions from L_1–2_ to L_5_–S_1_ on both sides were measured with the three‐dimensional (3D) reconstruction computed tomography (CT) images at a standardized mid‐pedicle plane, which usually corresponds to the narrowest cross‐section area of the LIVF^17^, ^18^. The purpose of current study was: (i) to investigate the changes in the LIVF dimensions, including foraminal area, height, and width, from neutral to 30° hyperextension position; (ii) to investigate at which level the LIVF dimensions have the biggest and smallest changes; and (iii) to investigate the relation between the changes in LIVF dimensions and side, sex, and age.

We hypothesized that the LIVF dimensions would decrease with the hyperextension of the lumbar spine in supine position, and the changes in LIVF dimensions would not be influenced by side, sex, and age.

## Materials and Methods

### *Inclusion and Exclusion Criteria*

A total of 35 healthy volunteers (18 men and 17 women) with a mean age of 28.9 ± 5.0 years were enrolled in this study. Inclusion criteria included the following PICOS principle: (i) Healthy subjects who has no previous spinal diseases and surgical history; (ii) underwent lumbar spine 3D CT in hyperextension position; (iii) underwent lumbar spine 3D CT in neutral supine position; (iv) foraminal area, height, and width at all lumbar foraminal levels; and (v) a retrospective study. Exclusion criteria were: (i) current or prior lower back pain or radiculopathy; (ii) history of spinal surgery; and (iii) anatomic abnormalities or other spinal disorders.

### *Ethical Approval*

Our study was approved by the institutional review board of our institute, and informed consent was obtained from all participants.

### *Evaluation Method*

**Figure 1:**
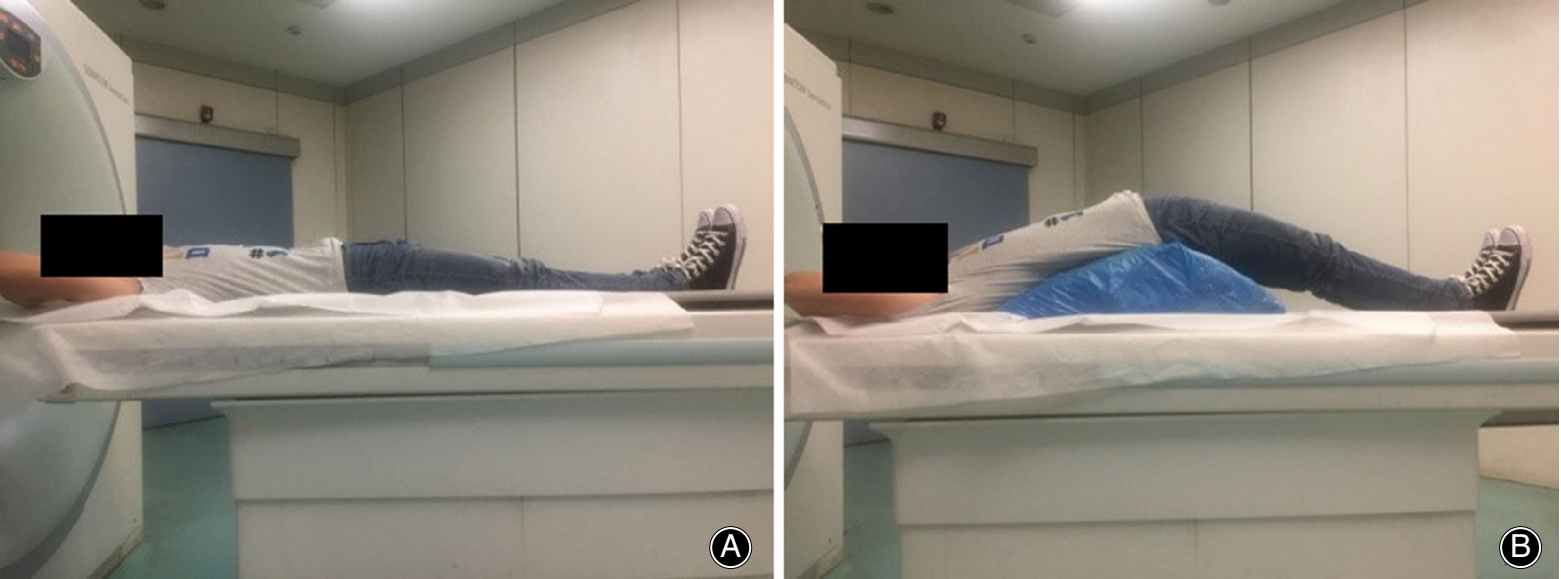
Body position of asymptomatic volunteers. (A) Conventional supine position. (B) Hyperextension supine position. Subject lay with a position mat under the pelvis at the level of the anterior superior iliac spine.

The lumbar spine was scanned using multislice spiral CT with a slice thickness of 0.6 mm (Siemens, Germany) in September 2018. In all subjects, five foramina from L_1–2_ to L_5_–S_1_ were evaluated on both sides. The subjects were first scanned in conventional neutral supine position. Then we placed a position mat under the pelvis at the level of ASIS so that the hip was hyperextended at 30° during CT scanning, to mimic the hyperextension supine position in the DAA THA (Fig. 1).The 30° angle was determined by protractor.

**Figure 2:**
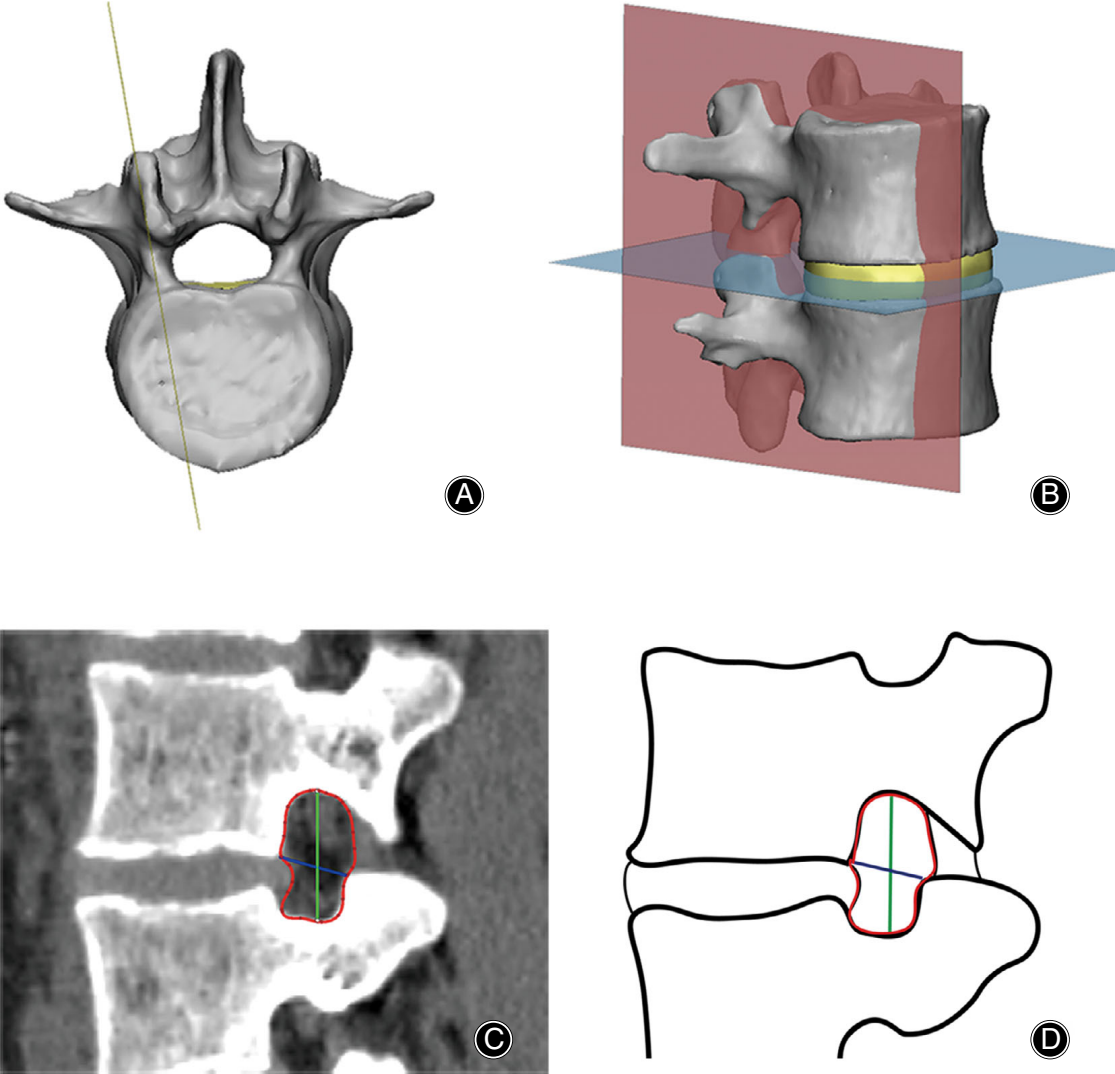
Pedicle–pedicle method for measurement of the lumbar intervertebral foramen. (A) The special sagittal slice was aligned along the midline of superior and inferior pedicles. (B) The slice was perpendicular to the disc space. (C) Foraminal area (red) was defined as the area bounded by the adjacent superior and inferior vertebral pedicles, the posterosuperior portion of the inferior vertebral body, the surface of the intervertebral disk posteriorly, the posteroinferior portion of the superior vertebral body, and the surface of the ligamentum flavum anteriorly. Foraminal height (green) was defined as the longest distance between the border of the superior and the inferior pedicle. Foraminal width (blue) was defined as the distance between the posteroinferior edge of the superior vertebrae and the anterior boundary of superior articular process. (D) Diagram of foraminal area (red), foraminal height (green), and foraminal width (blue).

All image data were saved in DICOM format, and were subsequently imported to Mimics 19.0 (Materialize, Leuven, Belgium) for preprocessing. Several previous studies have used 3D CT scans for the measurement of LIVF dimensions^9^, ^13^, ^17^. Among them, Rao *et al*.^17^ proposed a pedicle‐to‐pedicle method that could obtain a standardized snapshot of the foramen, and, thus, we adopted their method in our study. According to Rao’s description, the special sagittal slice was aligned along the midline of superior and inferior pedicles and perpendicular to the disc space by the “reslice” function of mimics (Fig. 2A,B). Then the dimensions of LIVF were obtained by the “measure” function of mimics.

### *Parameters*

#### *Foraminal Area*

The foraminal area was defined as the outline of the LIVF^10^. It is measured by the boundary of the adjacent superior and inferior vertebral pedicles, the posterosuperior portion of the inferior vertebral body, the posterior portion of the intervertebral disc, the posteroinferior portion of the superior vertebral body, and the anterior portion of ligamentum flavum^10^ (Fig. 2C,D). Previous studies report that foraminal area narrowing can result in nerve root compression in the lumbar region^10^, ^19^. We measured the foraminal area in neutral and hyperextension supine position, respectively, and the percentage changes of the foraminal area from neutral to hyperextension supine position were also calculated.

#### *Foraminal Height*

Foraminal height was defined as the longest distance between the boundary of the superior and inferior pedicle^10^, ^20^. It was measured from the most inferior aspect of the upper pedicle to the most superior aspect of the lower pedicle^10^, ^20^ (Fig. 2C,D). Because the anatomical morphology of the pedicle is fixed, the upper and lower movability range of the nerve root is determined by the distance between the adjacent pedicles^9^. We measured the foraminal height in neutral and hyperextension supine position, respectively, and the percentage changes of foraminal height from neutral to hyperextension supine position were also calculated.

#### *Foraminal Width*

Foraminal height was defined as the distance between the posteroinferior edge of the superior vertebrae and the anterior boundary of the superior articular process^13^. It was measured on the line through the posteroinferior corner of the superior vertebrae and vertical to the anterior surface of the opposing facet^13^ (Fig. 2C,D). Because the anatomical morphology of the pedicle is fixed, the anterior and posterior movability range of the nerve root is determined by the distance between the posteroinferior edge of the superior vertebrae and the anterior boundary of the superior articular process^9^. We measured the foraminal width in neutral and hyperextension supine position, respectively, and the percentage changes of foraminal width from neutral to hyperextension supine position were also calculated.

### *Statistical Analysis*

All parameters were expressed with mean ± SD. All statistical analysis was performed with SPSS 21.0 software (IBM, Armonk, USA). Two of the authors performed blinded measurements. One of the authors repeated measurements with 4‐week intervals. The reliability of intrarater and interrater measurements were assessed using intraclass correlation coefficients (ICCs), which can be interpreted as: <0.40 poor; 0.40–0.59 fair; 0.60–0.74 good; 0.75–1.00 excellent^21^. The Wilcoxon signed‐rank test was used to compare the LIVF dimensions measured in two positions. The paired *t*‐test was used to compare the percentage changes of LIVF dimensions between right and left sides, and the independent *t* test was used to compare the percentage changes between sexes. Multiple linear regression was used to evaluate the relationship between the percentage changes of LIVF dimensions and the subjects’ age and sexes. A *P*‐value less than 0.05 was considered significant difference.

## Results

### *Reliability*

The intraclass correlation was found to be 0.92 for foraminal area, 0.94 for foraminal height and 0.97 for foraminal width. The interclass correlation was 0.88 for foraminal area, 0.90 for foraminal height, and 0.91 for foraminal width. All measurements showed excellent ICCs.

### *Foraminal Area*

**Figure 3:**
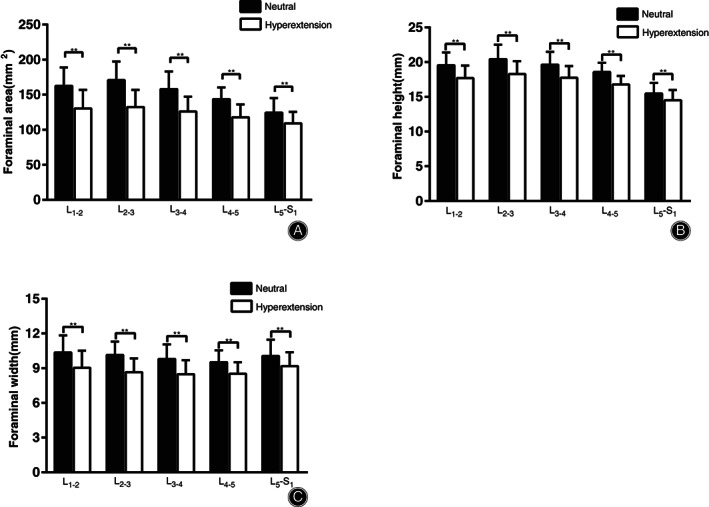
(A) Foraminal area of all lumbar intervertebral levels from L_1–2_ to L_5_–S_1_ in neutral and 30°hyperextension supine position. (B) Foraminal height of all lumbar intervertebral levels from L_1–2_ to L_5_–S_1_ in neutral and 30° hyperextension supine position. (C) Foraminal width of all lumbar intervertebral levels from L_1–2_ to L_5_–S_1_ in neutral and 30° hyperextension supine position. **Significant differences were found between two positions (*P* < 0.01).

The foraminal area varied significantly between the two positions at all levels (*P* < 0.05; Table 1, Fig. 3A). From neutral to hyperextension supine position, the foraminal area reduced by 32.2 ± 10.6 mm^2^ (20.1%) at L_1–2_, 38.5 ± 12.3 mm^2^ (22.6%) at L_2–3_, 32.0 ± 14.5 mm^2^ (19.9%) at L_3–4_, 25.7 ± 10.3 mm^2^ (18.1%) at L_4–5_, and 15.3 ± 6.8 mm^2^ (12.0%) at L_5_–S_1_ level, respectively.

**Table 1:** Changes in lumbar intervertebral foramen dimensions at different levels from neutral to hyperextension supine position (mean ± SD)

Location	Neutral	Hyper‐extension	Change		*P*
			Absolute value	%	
L_1‐2_					
Area (mm^2^)	162.4 ± 26.4	130.2 ± 26.8	32.2 ± 10.6	20.1 ± 6.4	<0.01
Height (mm)	19.6 ± 1.9	17.7 ± 1.8	1.9 ± 0.8	9.5 ± 3.7	<0.01
Width (mm)	10.4 ± 1.5	9.0 ± 1.5	1.3 ± 0.8	12.8 ± 7.4	<0.01
L_2‐3_					
Area (mm^2^)	170.8 ± 26.4	132.3 ± 24.6	38.5 ± 12.3	22.6 ± 6.6	<0.01
Height (mm)	20.4 ± 2.1	18.3 ± 1.9	2.2 ± 0.9	10.5 ± 3.9	<0.01
Width (mm)	10.1 ± 1.2	8.7 ± 1.2	1.5 ± 0.7	14.5 ± 7.9	<0.01
L_3‐4_					
Area (mm^2^)	158.0 ± 25.2	126.0 ± 21.2	32.0 ± 14.5	19.9 ± 7.7	<0.01
Height (mm)	19.6 ± 1.9	17.8 ± 1.7	1.9 ± 0.8	9.5 ± 4.0	<0.01
Width (mm)	9.8 ± 1.3	8.5 ± 1.2	1.3 ± 0.9	13.0 ± 8.8	<0.01
L_4‐5_					
Area (mm^2^)	143.4 ± 16.8	117.7 ± 18.3	25.7 ± 10.3	18.1 ± 7.1	<0.01
Height (mm)	18.6 ± 1.8	16.8 ± 1.2	1.8 ± 0.8	9.6 ± 4.3	<0.01
Width (mm)	9.5 ± 1.0	8.5 ± 1.0	1.0 ± 0.6	10.4 ± 6.2	<0.01
L_5_–S_1_					
Area (mm^2^)	124.3 ± 20.7	109.0 ± 16.6	15.3 ± 6.8	12.0 ± 4.5	<0.01
Height (mm)	15.5 ± 1.5	14.5 ± 1.5	1.0 ± 0.6	6.1 ± 3.8	<0.01
Width (mm)	10.1 ± 1.4	9.2 ± 1.2	0.9 ± 0.7	8.4 ± 6.4	<0.01

### *Foraminal Height*

The foraminal height varied significantly between the two positions at all levels (*P* < 0.05) (Table 1, Fig. 3B). From neutral to hyperextension supine position, the foraminal height reduced by 1.8 ± 0.8 mm (9.5%) at L_1–2_, 2.2 ± 0.9 mm (10.5%) at L_2–3_, 1.9 ± 0.8 mm (9.5%) at L_3–4_, 1.8 ± 0.8 mm (9.6%) at L_4–5_, and 1.0 ± 0.6 mm (6.1%) at L_5_–S_1_ level, respectively.

### *Foraminal Width*

The foraminal width varied significantly between the two positions at all levels (*P* < 0.05) (Table 1, Fig. 3C). From neutral to hyperextension supine position, the foraminal height reduced by 1.3 ± 0.8 mm (12.8%) at L_1–2_, 1.5 ± 0.8 mm (14.5%) at L_2–3_, 1.3 ± 0.9mm (13.0%) at L_3–4_, 1.0 ± 0.6 mm (10.4%) at L_4–5_ and 0.9 ± 0.7 mm (8.4%) at L_5_–S_1_ level, respectively.

### *Relationship between Percentage Changes of the Lumbar Intervertebral Foramen Dimensions and Side, Sex, and Age*

No significant difference was found between the changes in LIVF dimensions (foraminal area, height, and width) and the side and sex (*P* > 0.05) (Tables 2 and 3). The overall regression analyses for all three measurements were found to be not statistically significant (*P* > 0.05) (Table 4).

**Table 2:** Percentage changes of lumbar intervertebral foramen dimensions for the male and female (mean ± SD, %)

Location	Right			Left			All		
	Male	Female	*P*	Male	Female	*P*	Male	Female	*P*
L_1–2_									
Area	19.7 ± 6.3	20.3 ± 7.0	0.81	20.2 ± 6.5	20.4 ± 6.5	0.93	20.0 ± 6.3	20.3 ± 6.6	0.81
Height	9.7 ± 4.1	9.1 ± 2.9	0.58	10.1 ± 4.5	9.1 ± 3.1	0.43	9.9 ± 4.3	9.1 ± 3.0	0.33
Width	12.8 ± 7.7	12.6 ± 6.6	0.95	11.9 ± 8.6	14.0 ± 6.9	0.44	12.3 ± 8.0	13.3 ± 6.7	0.59
L_2–3_									
Area	22.0 ± 4.7	23.6 ± 8.2	0.49	21.9 ± 4.7	23.1 ± 8.4	0.62	22.0 ± 4.6	23.4 ± 8.2	0.39
Height	10.3 ± 3.1	10.7 ± 4.9	0.73	10.2 ± 3.1	10.8 ± 4.5	0.66	10.2 ± 3.1	10.7 ± 4.7	0.57
Width	13.1 ± 4.8	16.6 ± 9.9	0.20	12.4 ± 6.3	16.1 ± 9.5	0.19	12.7 ± 5.6	16.3 ± 9.6	0.06
L_3‐4_									
Area	20.8 ± 7.9	19.0 ± 7.4	0.49	21.1 ± 8.2	18.6 ± 7.6	0.35	21.0 ± 8.0	18.8 ± 7.4	0.24
Height	8.7 ± 3.8	10.3 ± 4.2	0.24	9.1 ± 4.1	9.9 ± 4.2	0.57	8.9 ± 3.9	10.1 ± 4.1	0.21
Width	15.1 ± 9.4	11.5 ± 7.5	0.23	14.5 ± 10.0	10.7 ± 8.1	0.23	14.8 ± 9.6	11.1 ± 7.7	0.08
L_4‐5_									
Area	18.2 ± 8.0	17.5 ± 6.1	0.75	18.4 ± 8.5	18.1 ± 5.9	0.91	18.3 ± 8.1	17.8 ± 5.9	0.76
Height	9.3 ± 4.3	9.8 ± 4.4	0.74	9.3 ± 4.2	9.9 ± 4.5	0.69	9.3 ± 4.2	9.9 ± 4.4	0.59
Width	10.8 ± 8.1	10.3 ± 3.7	0.83	11.8 ± 6.7	8.7 ± 5.6	0.15	11.3 ± 7.3	9.5 ± 4.7	0.23
L_5_‐S_1_									
Area	12.9 ± 3.8	11.2 ± 4.9	0.26	12.5 ± 4.6	11.1 ± 4.9	0.39	12.7 ± 4.2	11.2 ± 4.8	0.15
Height	6.4 ± 2.6	6.1 ± 3.9	0.78	6.9 ± 3.9	5.0 ± 4.6	0.18	6.7 ± 3.3	5.5 ± 4.3	0.21
Width	8.6 ± 6.9	8.3 ± 6.3	0.92	9.0 ± 6.7	7.6 ± 6.1	0.54	8.8 ± 6.7	8.0 ± 6.1	0.61

**Table 3:** Percentage changes of LIVF Dimensions for the left and right sides (mean ± SD, %)

Location	Male			Female			All		
	Right	Left	*P*	Right	Left	*P*	Right	Left	*P*
L_1‐2_									
Area	19.7 ± 6.3	20.2 ± 6.5	0.52	20.3 ± 7.0	20.4 ± 6.5	0.87	20.0 ± 6.5	20.3 ± 6.4	0.52
Height	9.7 ± 4.1	10.1 ± 4.5	0.37	9.1 ± 2.9	9.1 ± 3.1	0.97	9.4 ± 3.5	9.6 ± 3.9	0.51
Width	12.8 ± 7.7	11.9 ± 8.6	0.22	12.6 ± 6.6	14.0 ± 6.9	0.06	12.7 ± 7.1	12.9 ± 7.7	0.68
L_2‐3_									
Area	22.0 ± 4.7	21.9 ± 4.7	0.75	23.6 ± 8.2	23.1 ± 8.4	0.34	22.8 ± 6.6	22.5 ± 6.7	0.33
Height	10.3 ± 3.1	10.2 ± 3.1	0.75	10.7 ± 4.9	10.8 ± 4.5	0.97	10.5 ± 4.0	10.5 ± 3.8	0.88
Width	13.1 ± 4.8	12.4 ± 6.3	0.31	16.6 ± 9.9	16.1 ± 9.5	0.43	14.8 ± 7.8	14.2 ± 8.1	0.19
L_3‐4_									
Area	20.8 ± 7.9	21.1 ± 8.2	0.61	19.2 ± 7.4	18.6 ± 7.6	0.46	20.0 ± 1.3	19.9 ± 1.3	0.88
Height	8.7 ± 3.8	9.1 ± 4.1	0.35	10.3 ± 4.2	9.9 ± 4.2	0.20	9.5 ± 4.0	9.5 ± 4.1	0.93
Width	15.1 ± 9.4	14.5 ± 10.0	0.47	11.5 ± 7.5	10.7 ± 8.1	0.34	13.3 ± 8.6	12.7 ± 9.2	0.23
L_4‐5_									
Area	18.2 ± 8.0	18.4 ± 8.5	0.75	17.5 ± 6.1	18.1 ± 5.9	0.21	17.9 ± 7.0	18.2 ± 7.3	0.26
Height	9.3 ± 4.3	9.3 ± 4.2	0.94	9.8 ± 4.4	9.9 ± 4.5	0.75	9.6 ± 4.3	9.6 ± 4.3	0.78
Width	10.8 ± 8.1	11.8 ± 6.7	0.10	10.3 ± 3.7	8.7 ± 5.6	0.12	10.5 ± 6.3	10.3 ± 6.3	0.73
L_5_–S_1_									
Area	12.9 ± 3.8	12.5 ± 4.6	0.55	11.2 ± 4.9	11.1 ± 4.9	0.86	12.1 ± 4.4	11.8 ± 4.7	0.60
Height	6.4 ± 2.6	6.9 ± 3.9	0.41	6.1 ± 3.9	5.0 ± 4.6	0.10	6.2 ± 3.2	6.0 ± 4.3	0.62
Width	8.6 ± 6.9	9.0 ± 6.7	0.84	8.3 ± 6.3	7.6 ± 6.1	0.49	8.4 ± 6.5	8.3 ± 6.3	0.90

**Table 4:** Relationship between percentage changes of LIVF dimensions and sexes and age

Location	Area		Height		Width	
	Right	Left	Right	Left	Right	Left
L_1‐2_						
Sex	*t* = 0.21	*t* = 0.08	*t* = −0.55	*t* = −0.77	*t* = −0.10	*t* = 0.75
	*P* = 0.84	*P* = 0.94	*P* = 0.59	*P* = 0.45	*P* = 0.92	*P* = 0.46
Age	*t* = 1.1	*t* = 0.36	*t* = 0.04	*t* = −0.74	*t* = 1.1	*t* = 0.93
	*P* = 0.28	*P* = 0.72	*P* = 0.97	*P* = 0.47	*P* = 0.30	*P* = 0.36
L_2‐3_						
Sex	*t* = 0.69	*t* = 0.49	*t* = 0.38	*t* = 0.46	*t* = 1.32	*t* = 1.33
	*P* = 0.50	*P* = 0.63	*P* = 0.71	*P* = 0.65	*P* = 0.20	*P* = 0.19
Age	*t* = 0.53	*t* = 0.55	*t* = −0.10	*t* = −0.59	*t* = 0.62	*t* = −0.07
	*P* = 0.60	*P* = 0.59	*P* = 0.31	*P* = 0.56	*P* = 0.54	*P* = 0.95
L_3‐4_						
Sex	*t* = −0.69	*t* = −0.94	*t* = 1.18	*t* = 0.53	*t* = −1.20	*t* = −1.19
	*P* = 0.50	*P* = 0.35	*P* = 0.25	*P* = 0.60	*P* = 0.24	*P* = 0.24
Age	*t* = 0.16	*t* = 0.26	*t* = 1.44	*t* = 1.63	*t* = −1.00	*t* = −1.10
	*P* = 0.87	*P* = 0.80	*P* = 0.16	*P* = 0.11	*P* = 0.33	*P* = 0.28
L_4‐5_						
Sex	*t* = −0.31	*t* = −0.12	*t* = 0.40	*t* = 0.47	*t* = −0.27	*t* = −1.5
	*P* = 0.76	*P* = 0.91	*P* = 0.69	*P* = 0.64	*P* = 0.79	*P* = 0.14
Age	*t* = −0.19	*t* = −0.10	*t* = −1.63	*t* = −1.44	*t* = 1.60	*t* = 0.89
	*P* = 0.85	*P* = 0.93	*P* = 0.11	*P* = 0.16	*P* = 0.12	*P* = 0.38
L_5_–S_1_						
Sex	*t* = −1.14	*t* = −0.84	*t* = −0.35	*t* = −1.38	*t* = −0.12	*t* = −0.61
	*P* = 0.27	*P* = 0.41	*P* = 0.73	*P* = 0.18	*P* = 0.91	*P* = 0.55
Age	*t* = 0.15	*t* = −1.60	*t* = 1.77	*t* = 0.59	*t* = 0.40	*t* = 0.14
	*P* = 0.88	*P* = 0.12	*P* = 0.09	*P* = 0.56	*P* = 0.69	*P* = 0.89

## Discussion

In the current study, we investigated the LIVF dimensions including foraminal area, height, and width on the standardized mid‐pedicle slice and compared the parameters measured in conventional neutral supine and hyperextension supine position. The results confirmed our initial hypothesis that the LIVF dimensions were significantly decreased at all intervertebral foraminal levels with the hyperextension of the lumbar part. The changes in LIVF dimensions were largest at L_2–3_ level, and smallest at L_5_–S_1_ level. In addition, the percentage changes were not influenced by side, sex, and age.

Several *in vivo* studies have reported position‐dependent morphologic changes of LIVF. Generally, the LIVF dimensions increase in flexion and decrease in extension^11^, ^12^, ^13^, ^14^, ^15^. The reduction in LIVF dimensions could result in increased compression on the nerve root due to facet joint motion and ligamentum flavum bulging^16^. In some patients, the nerve root impingement caused by foraminal stenosis occurs in extension but is relieved in flexion^22^, ^23^. Hyperextending the hip joint is recommended to attain appropriate exposure of the proximal femur during the femoral preparation in patients undergoing DAA THA^3^, ^5^, ^6^; therefore, a special position might change the morphology of the LIVF and lead to more pressure on and potential injury of the spinal nerve root.

This study first investigated the changes in LIVF dimensions when subjects were placed in DAA‐specific hyperextension supine position. Our results showed similar decrease of the foraminal area to the previous studies at L_1_–L_5_ levels^11^, ^12^, ^13^, ^14^. However, the change in the foraminal area reached significant difference at the L_5_–S_1_ level, which was similar to the results in Singh’s and Schmid’s study^11^, ^14^ but different from those in Ren’s and Zhong’s study^12^, ^13^. The foraminal height and width also reached significant difference at the L_5_–S_1_ level in our study. These differences might be attributed to the different imaging modalities and measurement methods. Moreover, putting the mat under the subjects could place them in passive hyperextension position, and the kinematics in such a position might differ from the active extension position reported in previous studies.

In our study, the changes in LIVF dimensions were largest at L_2–3_ level, which was coincident with Ren’s study^12^. The changes were smallest at L_5_–S_1_ level, which was possibly due to different biomechanics at L_5_–S_1_ level compared to the upper levels. The sacrum is markedly curved and tilted backwards, and the first sacral vertebra articulates with the fifth lumbar vertebra at specific lumbosacral joint angle^19^. Thus, the L_5_–S_1_ intervertebral foramen is thought to be less mobile compared to upper levels in extension of the lumbar spine^11^, ^19^. However, the incidence of lumbar nerve root compression was found to be rare in L_2_ nerve root but common in L_5_ nerve root due to the increased ratio of the nerve root diameter to the foraminal area being lower compared to upper levels^16^. Based on the above reasons, attention should be paid to the decrease of LIVF dimensions at L_4–5_ and L_5_–S_1_ levels.

Although the percentage changes of foraminal width were slightly higher for the male subjects from L_2_–L_5_ levels, we did not detect a significant difference in LIVF dimensions between the sexes and sides at all levels. The results of the regression analysis showed no influence of side, sex, and age on the percentage changes in LIVF dimensions.

### *Limitations*

Some limitations to our study should be considered. First, the subjects involved in our study were mainly young and healthy volunteers; therefore, the changes might be different in older people and patients with lumbar spine diseases. Second, the patients were under general anesthesia during the operation, and the muscle relaxation effect possibly influenced the LIVF dimensions. The subjects in the current study were scanned in a conscious state, which might not accurately reflect the intraoperative changes of LIVF dimensions. The exact changes during the operation should be further investigated in future studies. Despite these limitations, our study still demonstrates a decrease in LIVF dimensions with the hyperextension of the lumbar spine in supine position.

### *Conclusion*

In this study, we observed that the LIVF dimensions, including area, height, and width, decreased significantly with extension of the spine in supine position. The biggest decrease of foraminal dimensions was at L_2–3_ level and the smallest at L_5_–S_1_ level. The changes in dimensions were not influenced by side, sex, and age. This study demonstrated the position‐dependent change of LIVF comparing the neutral supine position with the DAA‐specific hyperextension supine position. This is of great value for understanding and preventing the potential risk of nerve root injury during DAA THA.
